# Book review: The Logic of Madness: A New Theory of Mental Illness

**DOI:** 10.3389/fpsyg.2016.00946

**Published:** 2016-06-23

**Authors:** Claire M. Fletcher-Flinn

**Affiliations:** School of Psychology, University of AucklandAuckland, New Zealand

**Keywords:** psychiatry, mental illness, mental disorder, acute personality disorder, madness

Most of us believe that the mad are “mentally” ill, having a disease of the brain, either psycho-chemical or neurobiological in nature. This view is shared widely, by professionals in the field, and popularized in books, films, and other media. Professional diagnosis by psychiatrists of the various disorders is based on behavioral symptoms, as defined and classified by the American Psychiatric Association Diagnostic and Statistical Manual of Mental Disorders 5th Edition^1^ (*DSM*-*5*). Much of the behavior of those afflicted with a mental disorder, by definition, is not rational. But, could it be?

In his second book in a series (Figure 1), Matthew Blakeway contends that madness is indeed logical in structure. This assertion derives from his view (see Blakeway, 2014) that our brains are biological computers with algorithms for action based on emotional drivers. However, humans can out-think their biology by calculating an action to achieve an emotional goal (either current or future). This requires an understanding of the emotion involved and this is achieved by observing behavior. But what if that behavior has been modified by tactical deception (pretence)? This results in a misunderstanding of emotions, and leads to self-destructive actions. Humans perform tactical deception all the time. For example, how many of us would frequent a café or restaurant without being greeted with a friendly smile? The calculation is correct, but the input is faulty. Is the waitress *really* happy to see us, or would she rather be elsewhere? Blakeway places mental illness within the same framework, and argues that mental illness is simply self-destruction of a higher order of complexity.

**Figure 1:**
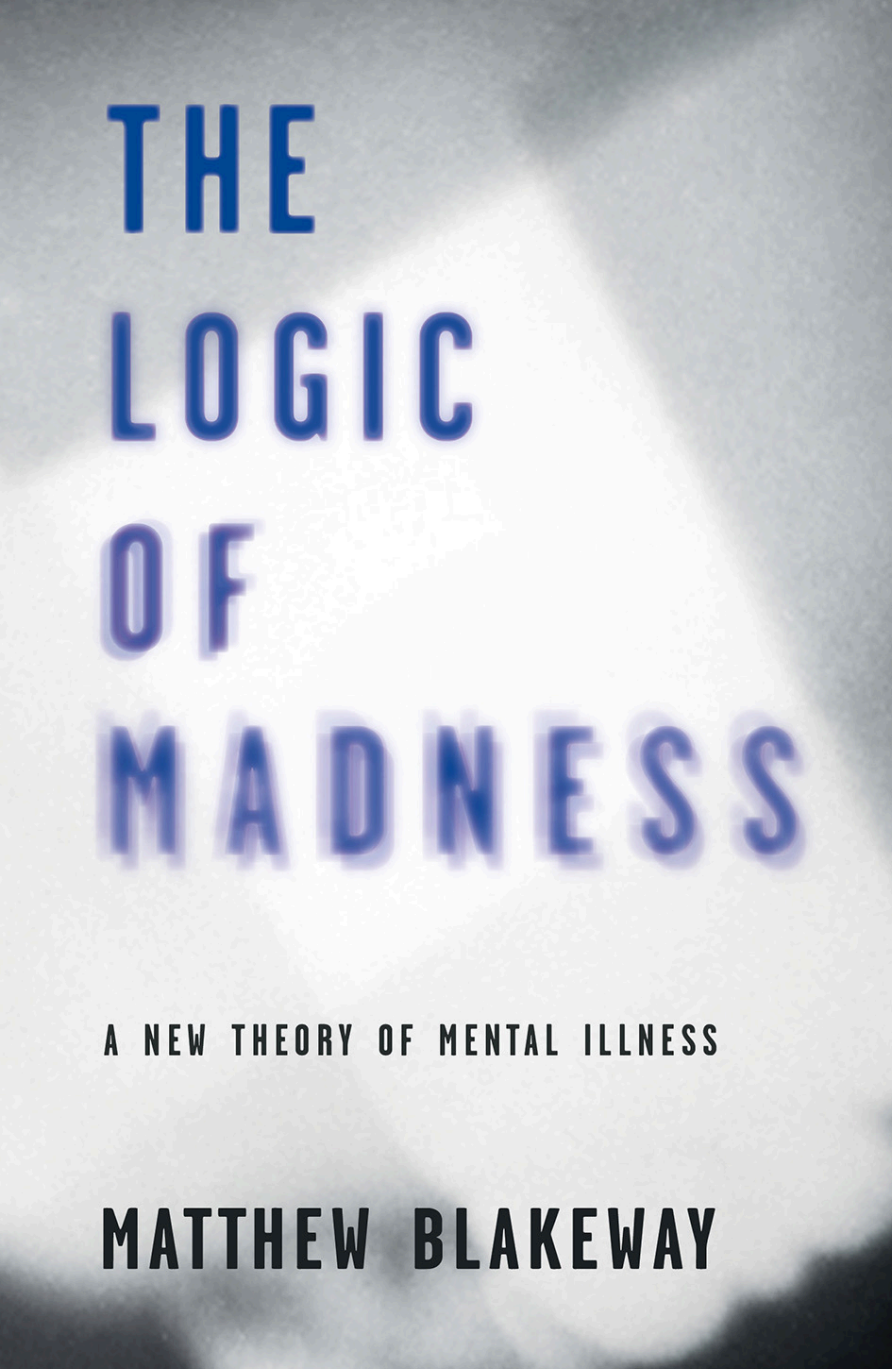
**Book Cover**.

Blakeway takes us on a historical journey of our understanding of madness from an affliction sent by God as punishment for our moral faults, to Freud's psychological theories of relationships and developmental frameworks, and the current understandings of neurological/biological causes with notions of “illness.” Cures for illness are dependent on causal theories. The point Blakeway makes is that we have no idea about the causal structure of the symptoms that are being treated. Psychopharmacological cures work, but we don't know why. They have an added bonus: drugs are cheap to administer compared with asylums, psychoanalysis, or psychotherapy. Szasz (1961) comes to mind here, although Blakeway believes madness is more than a myth, or social construct (Foucault, 2006). He contends that the particular behavioral pattern exhibited by the “mad” is dependent on the specific combination of inputs. It is rational behavior in response to a compound misunderstanding of various emotions.

The starting point of Blakeway's theory is a basic algorithm that converts an emotion into an action that optimizes biological fitness. Depending upon the circumstances, an action state is driven by the emotion having the highest calculated value. He divides emotions into four categories, basic survival (e.g., fear, hunger), reproductive (e.g., lust, jealousy), social (e.g., guilt, anger), and strategic (e.g., anxiety, regret). Most of these biologically derived emotions are shared with other animals, especially chimpanzees, although there is the question of whether other animals can perform tactical manipulation with *intent*. Humans can do so, which leads to misconceptions of emotion that become quite complex depending on whether we are performing the deception or someone else.

Blakeway separates mental disorders into three categories that overlap nicely with those of the *DSM-5*: compulsions (neuroses), impulsions (acute personality disorders), and delusions (psychoses), and he explains theoretically how and why they might occur. Set out in a series of tables and textual examples, he pulls apart the various emotional drivers and inhibitors that can lead to very different outcomes. He demonstrates how compulsions occur because two emotions get confused, and one becomes replaced by another. For example, if hunger (an emotional driver), was misinterpreted as shame (an inhibitor), then the appearance of food would lead to the suppression of the urge to eat, and in extreme cases, anorexia nervosa. As Blakeway notes, current medical opinion is that low self-esteem is a symptom of anorexia nervosa, but this could just as easily be the result of the systematic affectation of shame. This same mechanism can work in another way. If emotional inhibitors (e.g., shame) are confused, and act as drivers, this results in “libertine compulsions” where actions that should be suppressed (such as child molestation) become compulsions. Depression is an interesting case where all of the driving emotions “flatline,” so there is no desire to do anything. Like alcoholism, it is the aftermath (e.g., loss of relationships) that is the problem, not the action state itself.

Mental illness and criminality are closely connected. Prisons have an alarming number of people characterized with antisocial personality disorder, seen by Blakeway as a “proactive impulsion” resulting in the misidentification of shame as anger (a humiliation-revenge driver) resulting in an unprovoked attack on someone. Similarly, fear is the natural expressed emotion in situations of physical abuse, however, if flight is impossible and fear is suppressed, it can be mistaken for shame, leading to what Blakeway calls a “deviant anti-impulsion.” Thoughts can operate in the same way without the actual physical stimulus being present, and without biological brain dysfunctions. There is a strong correlation between dopamine and schizophrenia. Dopamine release is usually taken as causal, but what if it's the other way round? What if you can trick the brain into releasing dopamine, which is a pain inhibitor producing a feeling of euphoria? Being “away with the fairies” is perhaps not such a strange euphemism.

Blakeway has created a model of self-destructive complex action states that we commonly called “madness.” This is a provocative and radical new way of thinking, and one that deserves our attention. It opens the door to new therapies that specifically target the corrupted emotions. This could lead to better psychiatric care of the “mentally ill,” diminish drug therapies, and start to replace them with talk therapies combined with targeted cognitive re-training. These ideas should be given careful consideration if we are to provide the best help possible for those who need it the most.

## Author contributions

The author confirms being the sole contributor of this work and approved it for publication.

### Conflict of interest statement

The author declares that the research was conducted in the absence of any commercial or financial relationships that could be construed as a potential conflict of interest.

## Footnotes

^1^American Psychiatric Association (2013). *Diagnostic and Statistical Manual of Mental Disorders, 5th Edn.* Arlington, VA: American Psychiatric Publishing.
